# Interleukin-17 in the Nasal Polyps of Patients with Chronic Rhinosinusitis Compared to Healthy Controls: A Systematic Review

**DOI:** 10.1055/s-0046-1820090

**Published:** 2026-11-04

**Authors:** Paraskevi Karamitsou, Alexandros Poutoglidis, Kalliopi Domvri, Jannis Constantinidis, Theodora Papamitsou, Spyridon Gougousis, Konstantinos Porpodis

**Affiliations:** 1 Ear, Nose, and Throat (ENT) Department, East and North Hertfordshire Teaching NHS Trust, StevenageUnited Kingdom; 2 Laboratory of Anatomy and Surgical Anatomy, School of Medicine, Aristotle University of Thessaloniki, ThessalonikiGreece; 3 Laboratory of Histology-Embryology, School of Medicine, Aristotle University of Thessaloniki, ThessalonikiGreece; 4 1st Department of Otorhinolaryngology, School of Medicine, Aristotle University of Thessaloniki, ThessalonikiGreece; 5 Department of Otorhinolaryngology, Papanikolaou General Hospital, ThessalonikiGreece; 6 Pulmonary Department, School of Medicine, Aristotle University of ThessalonikiGreece

**Keywords:** rhinosinusitis, nasal polyps, interleukin-17, monoclonal antibodies, paranasal sinuses, nasal mucosa, inflammation, endoscopic sinus surgery, biological marker, treatment

## Abstract

**Introduction:**

Chronic rhinosinusitis with nasal polyps (CRSwNP) is an inflammatory disease of the sinonasal tract that significantly impacts quality of life. Novel therapies with monoclonal antibodies brought a paradigm shift to its treatment. Interleukin-17 (IL-17) is a known mediator of inflammatory pathways and its role in CRSwNP is debatable.

**Objectives:**

To evaluate the presence of IL-17 in the nasal polyps of patients with CRSwNP compared with subjects with healthy nasal mucosa.

**Data Synthesis:**

We conducted a systematic review according to the Preferred Reporting Items for Systematic Reviews and Meta-analyses (PRISMA) statement. The literature search was performed on the PubMed/MEDLINE, Google Scholar, and Cochrane Library. We identified 15 studies with a total of 785 patients with CRSwNP. The levels of IL-17 were increased in nasal polypoid tissue in most of the included studies.

**Conclusion:**

The current study suggests that anti-IL-17 treatments might effectively control CRSwNP. Further studies could assess their safety and efficacy for CRSwNP.

## Introduction

Chronic rhinosinusitis (CRS) is a common inflammatory disease of the sinonasal tract. Its subtype, CRS with nasal polyps (CRSwNP), accounts for 20 to 25% of cases, but there are cases that are characterized by chronic inflammation without nasal polyps (CRSsNP). 1 2 Polyps represent an inflammatory manifestation of the nasal mucosa. Another characteristic of some cases of CRSwNP is eosinophilic infiltration; this subtype is also associated with asthma and aspirin sensitivity. 3 4

Understanding of the immune system's response against pathogens that cross the mucosal barriers has contributed to the identification of the role of molecular pathways or endotypes in CRS. These endotypes seem to drive the inflammation, tissue remodeling, clinical phenotype, and final response to the treatment. 5 Chronic inflammation that leads to remodeling of the sinonasal tissues is mainly characterized by polyp formation, goblet cell hyperplasia, and epithelial barrier abnormalities. 5

In cases of CRS, mucosal barrier penetration results in a chronic inflammatory response that cannot be resolved. 5 The inflammatory process is featured by cell edema and the release of cellular contents. It is associated with a programmed cell apoptosis mechanism called pyroptosis. 6 However the caspase pathways activate this mechanism, 7 cytokines seem to play a crucial role as proinflammatory agents and inflammatory promoters. 8

The Th17 cells play a significant role in chronic allergic airway inflammation. 9 Interleukin-17 (IL-17) belongs to the group of inflammatory cytokines due to proinflammatory properties in activation of the autoimmune pathways, in cases of uncontrolled signaling. 10 Furthermore, Th17 cytokines affect the mucosal barrier structure and function of primary nasal epithelial cells. 9 Tissue swelling and increased nasal secretions after upregulation of matrix metalloproteinase-9 and mucin 5AC depends on the elevated IL-17 levels both in eosinophilic and neutrophilic patients with CRSwNP. However, the role of these cells in the pathogenesis of the studied condition is still debated. Our study aims to evaluate the presence of IL-17 in nasal polyps of patients with CRSwNP compared with subjects with healthy nasal mucosa and to determine its role in CRS pathogenesis.

## Literature Review

### Search Strategy

We examined patients with CRSwNP who underwent tissue biopsy compared with healthy controls for the levels of IL-17 in their specimens, according to the Patient, Intervention, Comparison, and Outcome (PICO) strategy. 11

We performed this systematic review following the Preferred Reporting Items for Systematic Reviews and Meta-analyses (PRISMA) statement and the recommendations of the Cochrane Collaboration. 12 Original comparative studies evaluating the levels of IL-17 in nasal tissue of patients with CRSwNP were selected. There was no restriction on the year of publication. A comprehensive electronic search in PubMed/MEDLINE, Google Scholar, and Cochrane Library was conducted between October 1st and 31st, 2023, to identify articles relevant to the research question.

The electronic databases were searched using the following free terms and medical subject headings (MeSH): (( *IL-17 in nasal polyps* ) OR ( *IL-17 in chronic rhinosinusitis* ) OR ( *IL-17 in CRSwNP* )).

### Selection Process

Inclusion and exclusion criteria were predetermined before the commencement of the literature search. Two authors (PK and AP) independently conducted the research and screened the studies according to the inclusion criteria. Disputes were resolved by consensus. All relevant articles identified for inclusion were also thoroughly hand-searched and evaluated for relevance.

Titles and abstracts of the potentially eligible studies were examined at first. Examination of the full text was performed for studies in which the correlation with the present research question was not clear only by reading the title and abstract. The reference list of the relevant studies was screened thoroughly for further identification of potentially eligible studies. Only articles written in English were evaluated for inclusion.

### Inclusion and Exclusion Criteria

The inclusion criteria for our systematic review were patients with CRSwNP who had undergone nasal tissue biopsy for evaluation of the IL-17 levels, presence of a control group with healthy nasal mucosa, and appropriate methodological analyses of the samples.

Furthermore, studies meeting at least one of the following criteria were excluded: animal studies, case reports, patients with nasal polyps without chronic rhinosinusitis, lack of a control group, conference papers with insufficient data, and studies with incomplete data.

### Data Collection Process

A data extraction template was prepared and used by both authors (PK and AP). The full texts of the eligible studies were obtained and for each study, the following were recorded: study characteristics (first author, date of publication, country of the first author's affiliation, study design, population with demographics) and primary endpoint.

### Risk of Bias Assessment

The Methodological Index for Non-randomized Studies (MINORS) tool was employed to assess the quality of the included studies of our systematic review. 13 This tool evaluates 12 different domains, with a score ranging from 0 to 2 (0 = not reported in the article, 1 = reported but inadequately, and 2 = reported adequately). The global ideal score is 16 for the noncomparative studies and 24 for the comparative ones. The current study did not require either ethical approval or informed patient consent because its design is that of a systematic review.

## Results

The initial search detected 1,561 studies. Additionally, 21 records identified through reference lists were evaluated for relevance. After excluding the duplicates, 1,499 records were eligible for assessment of their abstract. After thorough screening, 1,120 records were excluded, because they were not relevant to our criteria (cystic fibrosis, case reports, animal studies, noncomparative studies, lack of control group, serum studies).

As such, 363 articles were selected for a full-text extraction, and finally, 15 articles 14 15 16 17 18 19 20 21 22 23 24 25 26 27 28 that could answer the research question were included in the systematic review. The flow diagram of the literature search and selection process for appropriate studies is illustrated in Fig. 1 .

**Fig. 1:**
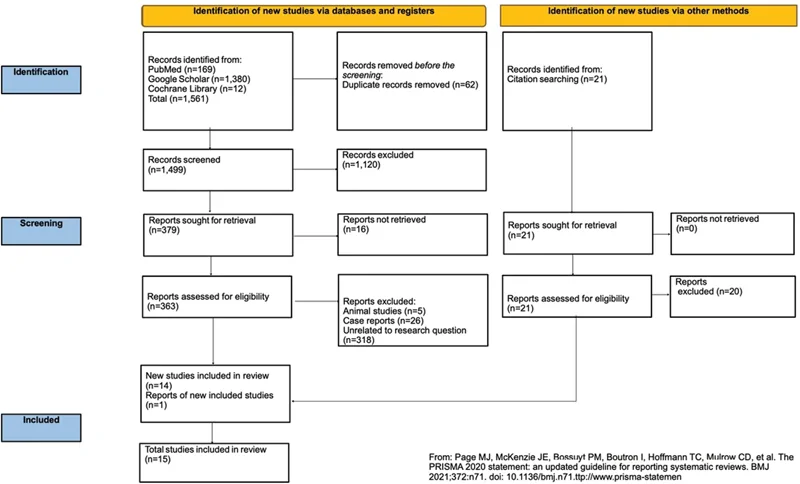
2020 Preferred Reporting Items for Systematic Reviews and Meta-analyses (PRISMA) flow diagram.

### Population and Demographics

A total of 785 patients with CRSwNP who underwent tissue biopsy of the nasal polyps and 529 healthy controls who underwent biopsy of their healthy nasal mucosa were included. The population of the included studies ranged from 19 to 232. All articles were published between 2009 and 2022. There were 12 studies 14 17 18 19 20 21 22 23 24 26 27 28 conducted in Eastern Asia, two, 15 25 in the United States, and one, 16 in Europe. Table 1 presents the characteristics of the included studies.

**Table 1:** The characteristics of the included studies

Study	Year	Country	Study design	Population (N)	Gender (male/female)
Shi et al. 14	2009	China	Observational (case-control)	35	21/14
Peters et al. 15	2010	USA	Observational (case-control)	56	28/28
Derycke et al. 16	2014	Belgium	Observational (case-control)	22	6/16
Shin et al. 17	2015	South Korea	Observational (case-control)	149	Not reported
Gong et al. 18	2017	China	Observational (case-control)	33	Not reported
Chen et al. 19	2018	China	Observational (case-control)	182	134/48
Kim et al. 20	2018	South Korea	Observational (case-control)	123	Not reported
Lin et al. 21	2018	China	Observational (case-control)	19	9/10
Yan et al. 22	2019	China	Observational (case-control)	284	Not reported
Zhang et al. 23	2019	China	Observational (case-control)	29	12/17
Kim et al. 24	2016	South Korea	Observational (case-control)	90	68/22
Smith et al. 25	2021	USA	Observational (case-control)	161	67/94
Lin et al. 26	2022	Taiwan	Observational (case-control)	66	43/23
Cheng et al. 27	2022	China	Observational (case-control)	70	Not reported
Li et al. 28	2022	China	Observational (case-control)	47	Not reported

### IL-17 Levels

The levels of IL-17 in the nasal polyp tissue of the patients were increased in 10 studies, 17 18 19 21 22 24 25 26 27 28 the same in two, 16 23 decreased in two, 14 20 and undetected in one study 15 when compared with the control group. Also, eight studies 14 15 17 19 21 23 27 28 used Enzyme-linked Immunosorbent Assay (ELISA) for IL-17 detection, three 16 20 22 used LuminexxMAP suspension array technology in a Bio-Plex 200 system (BioRad, MI, USA), one 24 used TaqMan assays (Life Technologies Korea, Seoul, Korea), one study 25 used Legendplex assay, one 26 used Bio-Plex Human Cytokine Group Assay kit (BioRad Laboratories, Hercules, CA, USA), and one study 18 used immunohistochemistry (rabbit anti-human IL-17 polyclonal antibody, Santa Cruz, USA) and real-time Polymerase Chain Reaction (PCR) (E.Z.N.A™ total RNA extraction kit, OMEGA, USA and reverse transcription kit, TAKARA, Japan). ( Table 2 ).

**Table 2:** Presence of IL-17 in nasal polyps compared with control healthy tissue

Study	Population (Control/CRSwNP)	Correlation of IL-17 in CRSwNP compared with the control group	Method of detection
Shi et al., 14 2009	11/24	Decreased	ELISA
Peters et al., 15 2010	18/38	IL-17 was not detected in either sample	ELISA
Derycke et al., 16 2014	7/15	Same	LuminexxMAP suspension array technology
Shin et al., 17 2015	27/122	Increased	ELISA
Gong et al., 18 2017	10/23	Increased	Immunohistochemistry & Real-time PCR
Chen et al., 19 2018	50/132	Increased	ELISA
Kim et al., 20 2018	45/78	Decreased	LuminexxMAP suspension array technology
Lin et al., 21 2018	8/11	Increased	ELISA
Yan et al., 22 2019	40/244	Increased	LuminexxMAP suspension array technology
Zhang et al., 23 2019	8/21	Same	ELISA
Kim et al., 24 2016	20/70	Increased	TaqMan assays
Smith et al., 25 2021	74/87	Increased	Legendplex assay
Lin et al., 26 2022	20/46	Increased	Bio-Plex Human Cytokine Group Assay
Cheng et al., 27 2022	17/53	Increased	ELISA
Li et al., 28 2022	22/25	Increased	ELISA

**Abbreviations:** CRSwNP, chronic rhinosinusitis with nasal polyps; ELISA, enzyme-linked immunosorbent assay; IL-17, interleukin-17; PCR, polymerase chain reaction.

### Method of Detection

Among the eight studies 14 15 17 19 21 23 27 28 that employed ELISA, five 17 19 21 27 28 reported increased IL-17 levels in CRSwNP compared with control tissue, one 23 reported no significant difference, one 14 reported decreased levels, and one 15 failed to detect IL-17. On the other hand, five 18 22 24 25 26 out of seven studies 16 18 20 22 24 25 26 using other detection techniques found increased IL-17 expression in CRSwNP tissue. This variability, particularly among ELISA-based studies, suggests a degree of heterogeneity. However, due to the lack of standardized quantitative data across studies, a formal statistical heterogeneity analysis could not be conducted, and a qualitative analysis was performed instead. Most studies showed an overall trend of increased IL-17 expression in CRSwNP. Additionally, the levels did not seem to be affected by the method of detection.

### Geographical Parameters

In nine 17 18 19 21 22 24 26 27 28 out of 12 Asian studies, 14 17 18 19 20 21 22 23 24 26 27 28 the levels of IL-7 in CRSwNP patients were increased compared with the control group. As for the three studies 15 16 25 conducted in the USA and Europe, the results seem to vary: one 16 study reported no significant difference, one 25 showed increased IL-17 expression, and one 15 failed to detect IL-17. In a geographical-based analysis for CRSwNP, it could be suggested that Th17 inflammation might be the dominant inflammatory process in Asian populations.

### Quality of the Included Studies

The included studies were mostly prospective, being evaluated with high-quality scores according to the MINORS quality assessment tool. Almost all of the studies had a clear aim and presented their results with validated statistical tools. Table 3 presents the evaluation of the included studies' quality.

**Table 3:** Quality assessment of the included studies according to the MINORS tool

Study	1	2	3	4	5	6	7	8	9	10	11	12	Score
Shi et al. 14	2	0	2	2	2	1	1	0	2	1	0	2	15
Peters et al. 15	2	0	2	2	2	1	1	0	2	2	1	2	16
Derycke et al. 16	1	0	2	2	2	1	1	0	1	2	1	2	15
Shin et al. 17	1	0	2	2	2	1	1	0	2	2	1	2	16
Gong et al. 18	2	0	2	2	2	0	0	0	2	2	2	2	16
Chen et al. 19	2	0	2	2	2	1	1	0	2	2	1	2	17
Kim et al. 20	2	0	2	2	2	1	1	0	2	2	2	2	18
Lin et al. 21	2	0	2	2	2	1	1	0	1	2	0	2	15
Yan et al. 22	1	0	2	2	2	1	1	0	2	2	2	2	17
Zhang et al. 23	2	0	2	2	2	1	1	0	2	2	2	2	18
Kim et al. 24	2	0	2	2	2	1	1	0	2	2	1	2	17
Smith et al. 25	2	0	2	2	2	1	1	0	2	1	1	2	16
Lin et al. 26	1	0	2	2	2	1	1	0	1	1	1	2	14
Cheng et al. 27	2	0	0	2	2	1	1	0	2	2	2	2	16
Li et al. 28	2	0	2	2	2	1	1	0	2	1	1	2	16

**Abbreviation:** MINORS, Methodological Index for Non-randomized Studies.

**Notes:** Points of evaluation in MINORS: 1. a clearly stated aim, 2. inclusion of consecutive patients, 3. prospective collection of data, 4. endpoints appropriate to the aim of the study, 5. unbiased assessment of the study endpoint, 6. follow-up period appropriate to the aim of the study, 7. loss to follow-up <5%, 8. prospective calculation of the study size, 9. an adequate control group, 10. contemporary groups, 11. baseline equivalence of groups, and 12. adequate statistical analyses.

## Discussion

Traditionally, CRSwNP is treated with nasal steroids and/or surgical interventions. Endoscopic sinus surgery (ESS) was considered the workhorse to alleviate patients with severe disease. However, recurrences after surgery do not seem to be the exception due to the inflammatory pathway activating CRS. 29

The identification of biological markers that promote the formation of nasal polyps led to a new era of immunologic agents targeting the inflammatory pathway. These novel treatments include monoclonal antibodies against specific cytokines or IgE proteins, reducing CRS's morbidity without the need for many surgical interventions. 30

Omalizumab, 31 32 an anti-IgE monoclonal antibody, dupilumab, 33 a monoclonal antibody blocking both IL-4 and IL-13 cytokines, and mepolizumab, 34 an IL-5 inhibitor, have been proven through clinical trials to be effective and safe choices for the management of CRSwNP.

Concerns for the role of IL-17 in the severity of CRS have been presented through the years. The Th17 cells are considered the main source of IL-17. However, CD8+ cells, γδ-T cells, some natural killer T (NKT) cells, TCRβ+ 'natural' Th17 cells, and myeloid cells can also produce this cytokine. 10 26 Also, IL-17 signaling induces the production of chemokines, proinflammatory cytokines, matrix metalloproteinases, antimicrobial peptides (AMPs), and inflammatory effectors. 10 Affection of the expression of matrix metalloproteinases can generate an abnormal degradation of the extracellular matrix leading to impaired cell repair and tissue remodeling. 35 An additional important factor of IL-17 function is its ability to act synergistically with other inflammatory stimuli. 10

In patients with CRSwNP, IL-17 increases the expression of MUC5B mucin. 36 Furthermore, it induces the secretion of other mediators of inflammatory diseases, such as IL-9 and granulocyte-colony stimulating factor (G-CSF). 37 In 2022, Wen et al. 38 demonstrated the major role of IL-17 along with other inflammatory factors, such as IL-6, IL-4, tumor necrosis factor-α (TNF-α), and others, in the severity of CRS.

In cases of noneosinophilic CRSwNP and CRSsNP, a mixed pattern of Th1 and Th17 inflammation with elevated levels of interferon-gamma (IFN-γ), TNF, and IL-17A has been detected. 26 A mutual regulation of the Th2 and Th17 pathways has been suggested in asthma. 39 Respectively, the interactive regulatory roles of Th2 and Th17 pathways have been found in CRSwNP. 40 It is already known that Th2-targeted therapeutic agents have been developed for patients with CRswNP.

Additionally, ixekizumab, bodalumab, and secukinumab are known anti-IL-17 agents used for severe plaque psoriasis and axial spondylitis. All these monoclonal antibodies are considered effective and safe for the control of previously-mentioned diseases according to a clinical trial by specifically targeting IL-17A or its receptor. 41 The use of agents with even increased specificity, such as Th2-related in combination with Th17-related agents, might permit a more successful management of CRSwNP. It could be assumed that their use might have been overlooked in the treatment. It is important to highlight that the inflammatory environment in CRSwNP is complex and heterogeneous. Therefore, while the mechanism of IL-17 inhibition supports a theoretical benefit, direct clinical evidence in CRSwNP is currently limited. Targeted clinical trials are essential to assess the safety and efficacy of anti-IL-17 therapies in this condition, as well as which patient subgroups may benefit the most. Thus, further research is required to evaluate the effectiveness of the anti-IL-17 agents in CRSwNP.

Our systematic review demonstrated overexpression of IL-17 in nasal polyps of patients with CRSwNP. The overexpression was recorded with various methods of detection and associated with the severity of the disease. However, nasal polyps may differ between Asian and European populations. 40 The Th17 pathway, in addition to the Th2, seems to be involved in the inflammatory process of CRSwNP in Asian populations. 40 Patients from Asian regions tend to exhibit higher IL-17 expression and greater neutrophilic infiltration. 42 In contrast, CRSwNP in European populations is predominantly characterized by Th2 inflammation, marked by eosinophilia, elevated IL-5 levels, and comparatively lower IL-17 expression. 40 42

Our evidence comes in agreement with Wang et al., 40 suggesting that different inflammatory pathways are responsible for CRSwNP among different populations. Genetic and environmental factors may influence cytokine expression profiles and immune responses, leading to differences in disease phenotypes. Certain populations may carry polymorphisms in cytokine-related genes, such as IL-17A and its receptors, which can modulate susceptibility to chronic inflammatory diseases and intensity of the immune response. 43 Additionally, factors such as allergen exposure, composition of the microbial flora, and regional patterns of antibiotic use may differentially affect mucosal immune activation across populations. 44 45

Several studies have demonstrated positive correlations between IL-17 levels and both patient-reported symptom severity and radiological disease extent in CRSwNP. Hussien et al. reported significant positive associations between serum IL-17 concentrations and both Sino-nasal Outcome Test (SNOT-22) symptom scores and Lund–Mackay computed tomography (CT) scores ( *p* < 0.001), as well as a significant association with polyp recurrence ( *p* = 0.005). 46 Similarly, a systematic review identified IL-17 as a biomarker correlated with disease severity, highlighting its potential as an indicator of overall disease burden and extent. 47 Moreover, tissue studies have shown that IL-17A expression is positively correlated with symptom severity, endoscopic grading, and radiological findings in patients with CRSwNP. 48 Therefore, IL-17 appears to be elevated in this cohort and associated with greater symptom burden and more extensive disease.

Our study has standard limitations. The included studies were mostly prospective. Although they were evaluated with high-quality scores, they were not randomized in design. The included patients were not consecutive cases and therefore a selection bias is possible. Furthermore, most studies did not report sample size calculations. Additionally, the subtypes of IL-17 were not analyzed and most studies considered the role of IL17A.

The subtypes of CRS among nations, races, and ethnicities do not follow proportional distribution. Most of our included studies are from Eastern Asia, making a geographical bias possible. Additionally, the relatively small sample of patients does not permit a safe geographical-based analysis for CRSwNP. Finally, the techniques used to detect and measure IL-17 levels varied across the included studies. Even among studies employing the same detection method, a degree of variability was observed, which may be attributed to differences in antibody specificity, detection sensitivity, assay protocols, and sample handling procedures. These factors can affect the comparability of results and contribute to methodological heterogeneity across studies.

## Final Comments

The IL-17 is a mediator of the inflammatory pathway of CRSwNP. It is found to be increased in nasal polyps of patients with this condition compared with the healthy tissue of the control group. The development of inhibitors against IL-17 may theoretically have a positive impact on CRSwNP, such as the anti-IgE, anti-IL-4, anti-IL-5, and anti-IL-13 already tested agents. Omalizumab, dupilumab, and mepolizumab brought a paradigm shift in the treatment of CRSwNP. Anti-IL-17 agents are already used against other diseases, such as plaque psoriasis and axial spondylitis, and may be tested for this disease. Our systematic review suggests that anti-IL-17 agents could be evaluated for their potential use in CRSwNP.
